# The Lack of High-quality Educational Resources about Adhesive Capsulitis on YouTube

**DOI:** 10.1055/s-0044-1785465

**Published:** 2024-04-10

**Authors:** Ali Yüce, Volkan Gür, Mustafa Yerli, Abdulhamit Misir

**Affiliations:** 1 Departamento de Ortopedia e Traumatologia, Prof. Dr. Cemil Taşcıoğlu City Hospital, Istambul, Turquia; 2 Departamento de Ortopedia e Traumatologia, Mengücek Gazi Education and Research Hospital, Erzincan Binali Yıldırım University, Erzincan, Turquia; 3 Clínica particular, Istambul, Turquia

**Keywords:** adhesive capsulitis, bursitis, video recording, social media, internet

## Abstract

**Objective** The advent of the Internet has provided new, easily accessible resources for patients seeking additional health information. Many doctors and healthcare organizations post informative videos on this platform, and nearly all patients are looking for videos online for a second opinion.

**Methods** The phrases "frozen shoulder," "frozen shoulder treatment," "adhesive" capsulitis, and "adhesive capsulitis treatment" were entered into YouTube's search bar for a normal inquiry. The informativeness and overall quality of the adhesive capsulitis videos were rated using three separate scales.

**Results** The mean and standard deviation values of the scoring systems were JAMA 1.25 ± 0.51, DISCERN 39.4 ± 13.4, GQS 2.83 ± 0.96 and ACSS 7.43 ± 4.86, respectively. Number of views, rate of views, and likes all had a positive correlation with Global Quality Score (GQS), as did DISCERN and ACSS. There was no statistically significant difference between the median JAMA, GQS score and Discern Criteria values according to the video source/uploader (p > 0.05).

**Conclusion** YouTube videos on adhesive capsulitis, thus, need to be of higher quality, reliability, and instructive quality. There is a need for reliable videos about adhesive capsulitis, with instructional and high-quality cited.

## Introduction

The advent of the Internet has provided new, easily accessible resources for patients seeking additional health information. 1 When it comes to broad Internet searches, YouTube is just second to Google in popularity. Patients, however, are becoming more inclined to it as a means of learning about available healthcare options. 2 Many doctors and healthcare organizations post informative videos on this platform, and nearly all patients are looking for videos online for a second opinion. YouTube is not a peer-reviewed platform, thus this development raises questions about the reliability of the information presented in its medical-related videos. 3 Adhesive capsulitis, also known as frozen shoulder, is a common shoulder problem that manifests with progressive loss of glenohumeral motion with pain. 4 This disease is one of the most common musculoskeletal problems seen in orthopaedics. This condition is quite prevalent in the orthopaedic population. Despite the prevalence of this problem and the advancements in shoulder surgery, many questions remain about the optimal course of therapy. 5 With these unknowns, patients with adhesive capsulitis will likely use YouTube to explore treatment options.

Several studies have shown evidence that the educational quality of YouTube videos dealing with orthopaedic diseases is inadequate. 1 2 3 6 7 8 Only one study in the literature examines youtube videos related to adhesive capsulitis. 9 The results of this study were consistent with those of other research. However, only videos that were relevant to a search keyword were used in their study. 9 Our goal with this study was to examine the informativeness and overall quality of these videos by expanding the search phrases adhesive capsulitis patients might use to find them on YouTube. As with other studies in the literature, we assumed that these videos' quality and instructional quality needed to be improved.

## Materials and Methods

On February 18, 2022, using Google Chrome (version 92.0.4515.159-64 bit) with the cache and cookies emptied, a search was performed on YouTube's database. Subjects included "frozen shoulder," "frozen shoulder treatment," "adhesive capsulitis," and "adhesive capsulitis treatment." The top 50 videos for each search keyword, as chosen by YouTube's algorithm based on "relevance," were included; this yielded a total of 200 videos for analysis. 10 Videos were considered for inclusion if they met the following criteria: they were in English, their principal subject was about frozen shoulder, and the audio and visual quality were satisfactory. Videos were excluded if they were repetitive, had no dialogue, were in a language other than English, were not about adhesive capsulitis, or were categorized as news, drama, or satire. There was no maximum duration for videos, and compilations of numerous episodes were counted as a single work. A YouTube ^®^ account was set up for the research, and once duplicates were eliminated, a complete list of video URLs was compiled. Only 173 videos were included for the study due to the exclusion of 26 that were considered to be repetitive and one that were in a language other than English.

For each included YouTube video, the following attributes were recorded: (1) title, (2) video duration, (3) views, (4) video source/uploader, (5) content type, (6) days since upload, (7) view rate (views/day), and (8) likes. The authors and uploaders of the videos were classified into seven groups: (1) academic (related to authors or uploaders affiliated with research groups, universities, or colleges), (2) physician (related to independent physicians or groups of physicians without research or academic affiliation), (3) non-physicians (healthcare workers other than licensed medical doctors), (4) trainer, (5) medical source (content or animations from health websites), (6) patient, and (7) commercial. The content types were categorized as: (1) exercise education, (2) disease-specific information, (3) patient experience, (4) surgical technique or approach, (5) non-surgical management, and (6) advertising.

**Fig. 1:**
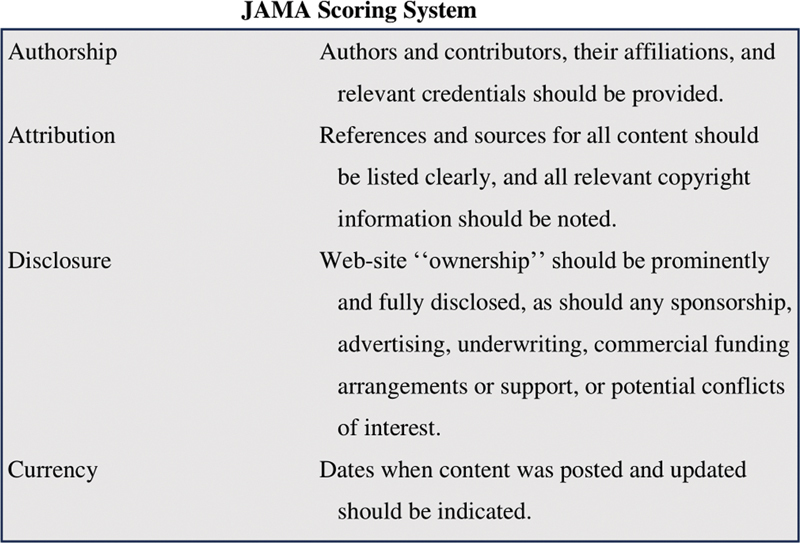
JAMA criteria.

The criteria published in the Journal of the American Medical Association (JAMA) were used to evaluate the accuracy and reliability of the videos ( Fig. 1 ). 11 Four factors, each weighted at 1, provide a generic evaluation of the credibility of the cited material. Accuracy and reliability are best represented by a score of 4, whereas a score of 0 shows the opposite. These criteria have been used extensively in the literature to assess the reliability of online resources, despite the fact that they have not been validated. 10 12

**Fig. 2:**
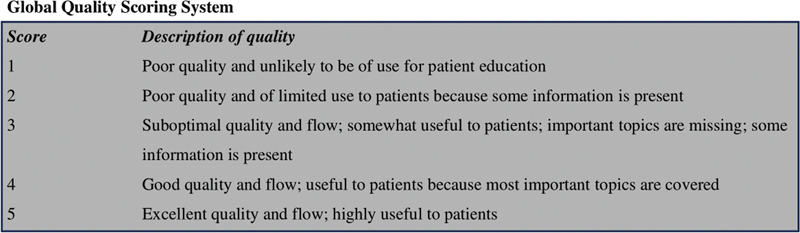
Global Quality Score.

**Fig. 3:**
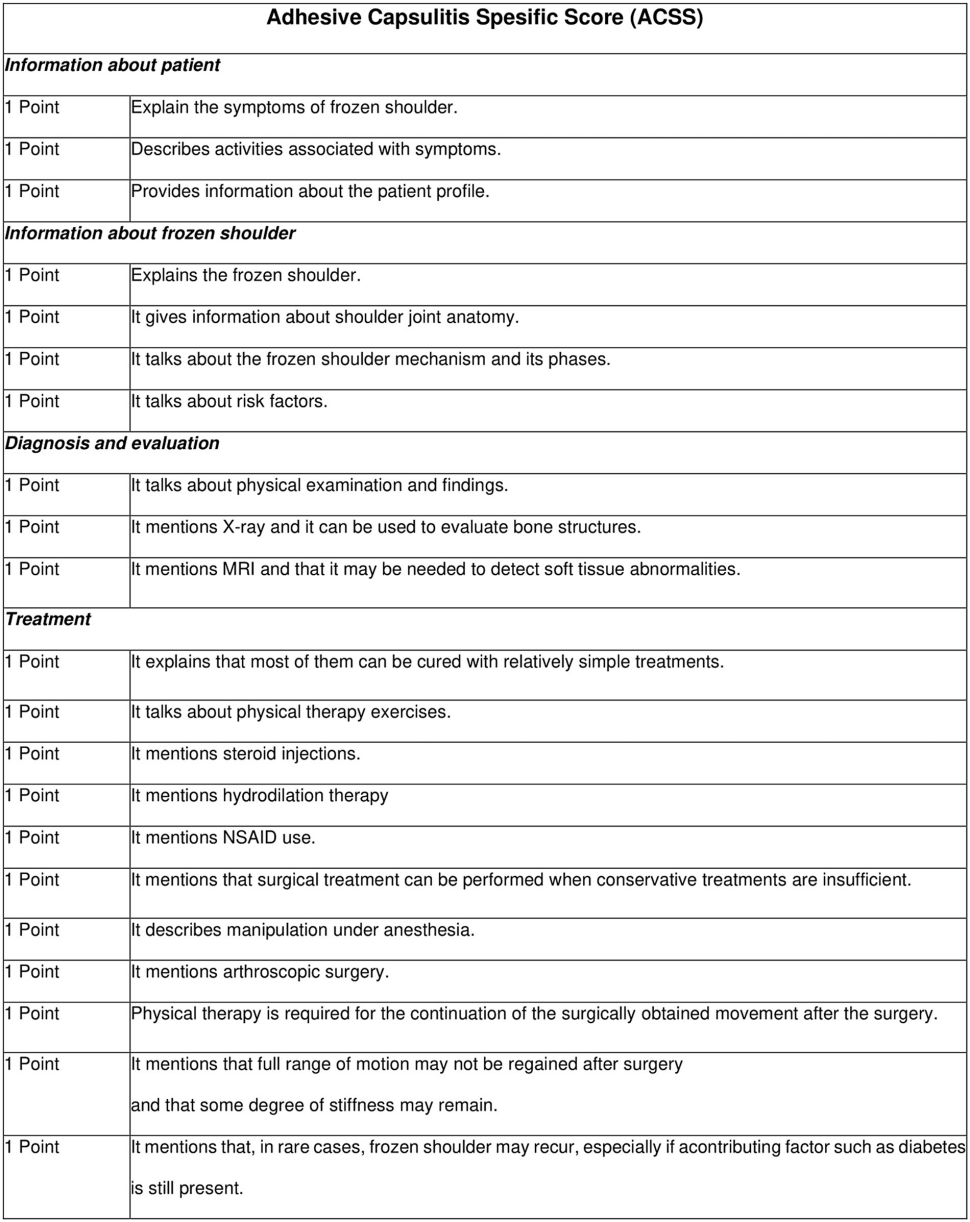
Adhesive Capsulitis Specific Score.

**Fig. 4:**
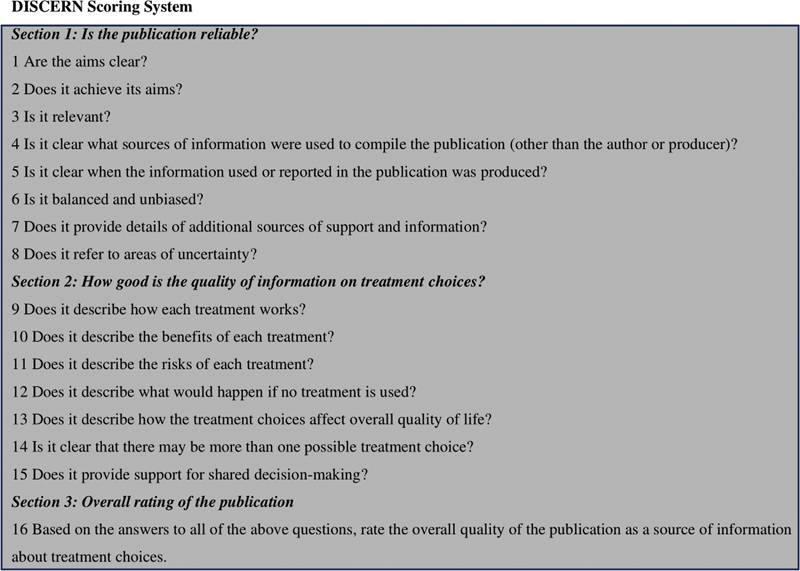
DISCERN score.

Three different scales were used to rate the educational value and quality of the adhesive capsulitis videos. Five factors are used to calculate the Global Quality Score (GQS) for educational content ( Fig. 2 ). 10 13 Quality education is represented by a maximum possible score of 5. The "Adhesive Capsulitis Specific Score" (ACSS) was developed for data pertaining to adhesive capsulitis, with its foundations on the recommendations made public by the American Academy of Orthopaedic Surgeons ( Fig. 3 ). The ACSS is a 21-item questionnaire that assesses information about patient presentation and symptoms, adhesive capsulitis in general, diagnostic and assessment procedures, and available treatment choices. Higher quality is represented by a higher score up to a maximum of 21. Very good (21 points), good (16 points), moderate (12 points), poor (8 points), and very poor (4 points) were the range of possible ACSS ratings. 10 13 The DISCERN score was created in Oxford, United Kingdom to evaluate the quality of health-related written materials. It consists of 16 questions, each of which is given a score between 1 and 5, giving a possible total of 6 to 80 ( Fig. 4 ). 14 Poor (16–28 points), poor (29–41 points), fair (42–54 points), good (55–67 points), and excellent (68–80 points) are the quality categories.

The videos included in the study were determined by the non-observer author and presented to the observers in a table format. The videos were examined and scored blindly by two observers who had been trained in pre-evaluation scoring using DISCERN, GQS, JAMA, and ACSS. The Interclass Correlation Coefficient (ICC) was used to determine the level of agreement across observers, with values below 0.5 indicating low reliability, between 0.5-0.75 suggesting moderate reliability, between 0.75 and 0.9 indicating good reliability, and above 0.9 indicating excellent reliability.

IBM SPSS Statistics version 20 was used for the data analysis. Continuous data were summarized as means and standard deviations while categorical data were summarized as percentages and relative frequencies. The numbers were rounded to one decimal place.Video reliability as well as quality were compared among video sources and content using either one-way analysis of variance (ANOVA) or Kruskal-Wallis tests, depending on the data distribution. Differences between groups were examined using the Mann-Whitney U test for statistical significance. The level of agreement between the reviewers was determined using the Interclass Correlation Coefficient (ICC). Spearman's rank correlation coefficient was used to examine correlations between evaluations of videos' usefulness and their technical characteristics. Statistical significance was assumed when the p-value was less than 0.05.

## Results

The averages of the features of the videos included in the study were: video duration 16.73 ± 123.09 minutes, number of views 264431.7 ± 617136.8, number of days after uploading 1537.95 ± 1159.3 days, view rate 269.75 ± 867.91 and number of likes 3826.78 ± 11595.45. Video source/uploader distribution 12 (6.9%) academic, 72 (41.6%) doctors, 71 (41%) non-physicians, 1 (0.6%) trainer, 13 (7.5%) medical sources, 2 (1.2%) were patients, and 2 (1.2%) were commercial. Looking at the contents of the videos, 44 (25.4%) were exercise training, 112 (64.7%) disease-specific information, 3 (1.7%) patient experience, 11 (6.4%) surgical technique/approach, and 3 (1.7%) included non-surgical management.

According to the JAMA criteria, 95.9% of the videos were rated 2 points or less. According to GQS, 27.7% of videos were rated 2 points or less. According to DISCERN criteria, 38 (21.9%) of the videos were very poor, 47 (27.2%) were poor, 62 (35.9%) were fair, 22 (12.7%) were good, and 4 (2.3%) were very good was evaluated. According to ACSS, 3 (1.7%) of the videos were very good, 31 (17.9%) good, 37 (21.4%) fair, 40 (23.1%) bad, and 62 (35.9%) vide rated very bad. The mean and standard deviation values of the scoring systems were JAMA 1.25 ± 0.51, DISCERN 39.4 ± 13.4, GQS 2.83 ± 0.96 and ACSS 7.43 ± 4.86, respectively. There were positive correlations between the number of views and GQS, between view rate and GQS, and between likes and GQS, DISCERN and ACSS (r:0.364, p < 0.001; r:0.414, p < 0.001; r:0.458, p < 0.001; r:0.265, p < 0.001; r:0.168, p < 0.027; respectively). There was no statistically significant difference between the median JAMA, GQS score and Discern Criteria values according to the video source/uploader (p > 0.05). The values of the scoring systems according to the video source/uploader are summarized in Table 1 .

**Table 1:** Mean and standard deviation values of scores by video source/Uploader

	Academic	Physician	Non-physician	Medical source
	Mean ± SD	Mean ± SD	Mean ± SD	Mean ± SD
JAMA	1.95 ± 1.15	1.19 ± 0.38	1.19 ± 0.41	1.26 ± 0.33
GQS	3.66 ± 0.65	2.45 ± 1	3.14 ± 0.75	2.96 ± 0.87
DISCERN Criteria	57 ± 14.37	36.74 ± 13.82	40.02 ± 10.37	40.88 ± 11.73
ACSS	13.45 ± 4.55	7.28 ± 5.13	9.53 ± 4.7	3.5 ± 3.53

A statistically significant difference was found between the median ACSS values according to the video source/uploader (p = 0.013). Here, the difference was seen between the ACSS median values of those whose video upload source was an instructor and those who were a medical source. The median ACSS value of the video upload source was 5, while the median PPIS value was 9 for the medical source ( Table 2 ). No statistically significant difference was found between the median values of JAMA, GQS score, Discern Criteria, and ACSSS values according to content type ( Table 3 ).

**Table 2:** Comparison of scores by video source/Uploader

				Video source/Uploader				
	Academic	Physician	Non-physician	Trainer	Medical source	Patient	Commercial	P-value *
	Mean(min-max)	Mean(min-max)	Mean(min-max)	Mean(min-max)	Mean(min-max)	Mean(min-max)	Mean(min-max)	
JAMA	1.5 (1-4)	2 (1-3)	1,5 (1-4)	1 (1-3)	2 (1-3)	1 (1-1)	1 (1-4)	0.081
GQS	2 (1-4)	2.8 (1-5)	2.5 (1-5)	2.5 (1-4)	3 (1-5)	1.8 (1-2.5)	2.5 (1-4)	0.715
DİSCERN Criteria	24 (16-47.5)	36 (14.5-56)	34 (15.5-55)	38 (16-63.5)	42 (23.5-54)	22 (14-30)	34 (20-46)	0.065
ACSS	5 (2-10) ^ab^	6,3 (1.5-17.5) ^ab^	6 (1-18) ^ab^	5 (1-13.5) ^a^	9 (5-14.5) ^b^	3 (2-4) ^ab^	9 (4-16) ^ab^	0.013

* Kruskal Wallis test ^a-b^ : There is no difference between groups with the same letter.

**Table 3:** Comparison of scores by content type

	Content type					
Exercise education	Disease-specific information	Patient experience	Surgical technique or approach	Non-surgical management	Advertising	P-value *
Mean(min-	Mean(min-	Mean(min-	Mean(min-		Mean(min-	
max)	max)	max)	max)	Mean(min-max)	max)	
JAMA 1.5 (1-3)	2 (1-4)	1 (1-1)	1.5 (1-4)	1.5 (1-3.5)	1 (1-4)	0.191
GQS 3 (1-4)	2.5 (1-4.5)	2.5 (2.5-2.5)	3 (1-5)	2.5 (1-5)	3 (1-4)	0.934
DISCERN Criteria 37.8 (25-63.5)	36.5 (15-55)	30 (30-30)	33 (14.5-56)	35 (14-54.5)	35 (20-46)	0.483
ACSS 5 (1-9)	7 (1.5-18)	4 (4-4)	6 (2.5-12.5)	5.5 (1-14.5)	9.5 (4-16)	0.051

* Kruskal Wallis Testi.

## Discussion

This study's essential findings are according to the JAMA criteria, 95.9% of the videos were rated 2 points or less. According to GQS, 27.7% of videos were rated 2 points or less. According to DISCERN criteria, 49.1% of the videos were evaluated as very poor or poor. According to the ACSS, 59% of the videos were rated as bad or very bad. These findings are similar to those of Tang et al., 9 which evaluated the educational and quality of adhesive capsulitis videos. This study has the feature of evaluating video reliability with JAMA scoring and evaluating more search terms and adhesive capsulitis videos that patients can search on YouTube. Another feature of this study is that there is no restriction on the duration of the video. Because as the duration of the videos increases, their information and educational content increase. 8 Failure to evaluate long videos may affect the research results by excluding highly educational videos. As a result of the comprehensive evaluation, this study concluded that the reliability, quality, and educational level of YouTube videos related to adhesive capsulitis needed to be improved.

Videos uploaded to YouTube do not go through an evaluation process. 3 For this reason, the number of likes and views of the videos can create a quality video perception in patients and cause misinformation. 15 As a result, the number of views of videos that are thought to be beneficial for patients may be less. 16 17 In this study, between the number of likes and the scoring; and there was a positive correlation between the number of views and GQS. These findings show that patients tend to watch better quality videos of adhesive capsulitis and like the ones that are highly educational. Our findings can be interpreted as adhesive capsulitis patients prefer videos that are educational and of high quality, but the number of these videos is insufficient.

The instruction for YouTube videos may vary depending on the video uploader and source. 18 Koller et al., 18 in their study evaluating videos about hip arthritis, found academic and doctor- sourced videos to be more educational. However, in this study, doctors or academic sources did not provide more educational information than other uploaders. Videos prepared for commercial purposes with commercial concerns may have negative consequences on the treatment of patients. 19 The major cause for poor videos might be related to commercial concerns. Given that most films are made in accordance with the provider's practice and there is no doctor-patient liability obligation, most providers may feel free to advise viewers about only particular parts of the condition and treatment methods. 3 This may cause patients with adhesive capsulitis to claim that the only treatment method offered is the right option and to request the wrong treatment. The solution to this situation may be to prepare patient information platforms without commercial concerns and to direct patients to these platforms.

Young patients use many social media platforms other than youtube to learn about their disease. 20 Artificially intelligent conversational agents (or "chatbots") have showed promise as direct patient engagement and tools for education, and Chat GPT is one such example. 21 These days' AI algorithms that deal with natural language are made to take in data that isn't neatly organized or standardized, and then provide results that sound human. These algorithms draw on a big corpus of previously written material by humans to create answers that have a high probability of matching the user's query. Chatbots have the potential to enhance medical care by supplying instantaneous answers to patient concerns, but because they are trained on language patterns rather than objective databases, they run the risk of giving patients erroneous but appearing reliable answers. 22 In order to learn more about the capacity and educational value of the information that patients access about frozen shoulder on the internet, more information can be obtained through studies examining frozen shoulder data on different social media platforms. In addition, there is a need to evaluate the information that chatbots provide to patients about frozen shoulder. Considering these data, artificial intelligence models can be trained by doctors. In this way, slideshows and videos that provide accurate and reliable information to patients can be prepared with artificial intelligence support. It can be made available to patients.

This study has some limitations. Videos are continuously being added to YouTube, making it a dynamic platform. It also offers personalized videos using artificial intelligence. Therefore, the videos watched in searches may only partially reflect the videos presented to patients. We used internet provider software with cleared cookies and history to minimize personalized video presentation. However, previous studies have also used this method. 12 23 Again, using only English videos and searching only from one location may change the properties of the evaluated videos. Artificial intelligence can offer different videos according to countries and locations. Different non-English search terms and videos may have different informational content. Only the first 50 videos were evaluated for each search term. The evaluated videos represent a small fraction of the videos associated with adhesive capsulitis. Findings may change as the numberof videos evaluated increases. However, this method has been used before. 10 12 At the same time, although we evaluated the adhesive capsulitis videos by expanding the search terms in this study, we assume that we obtained similar data to the findings of Tang et al. 9

The internet has made it easier than ever to access information on any topic imaginable. However, this also means a lot of misinformation and disinformation is available online. This can be a problem, as people may not be able to tell the difference between reliable and unreliable information. One way to address this problem is to filter information on the internet. This can be done by using software that identifies and blocks harmful or misleading content. However, it is important to note that no filtering system is perfect, and some false or misleading information may still slip through the cracks. Another way to address the problem is to educate people on critically evaluating information. This includes teaching people how to identify reliable sources of information, spot bias, and assess the quality of evidence. It is also important to be aware of the limitations of the internet. The internet is a vast and ever-changing resource, and it can be difficult to keep up with all the new information being published. This means that it is important to be skeptical of information that you find online, and to always do your own research before drawing any conclusions. Here are some tips for evaluating information on the internet: Consider the source of the information. Is it a credible website or organization? Look for evidence to support the claims being made. Are there any studies or statistics cited? Be aware of bias. Is the information coming from a biased source, such as a political party or a special interest group? Use your common sense. If something sounds too good to be true, it probably is. By following these tips, you can help ensure that you get accurate and reliable information from the internet.

Considering these findings, there is a need for educational and high-quality educational videos to inform patients. There should be clear and high-quality videos that deal with frozen shoulder as a whole, prepared by shoulder surgeons and their associations. These videos should be uploaded to public sites and patients should be directed to these videos. While preparing these videos, they can benefit from the information in https://www.mayoclinic.org/diseases - conditions/frozen-shoulder/symptoms-causes/syc-20372684, https://www.healthline.com/health/frozen-shoulder and https://orthoinfo.aaos.org/en/diseases - -conditions/frozen-shoulder/. In addition, by training artificial intelligence software on this disease, many videos with high quality content on frozen shoulder can be prepared quickly and effectively. It can be made available to patients.

## Conclusion

YouTube videos on adhesive capsulitis, thus, need to be of higher quality, reliability, and instructive quality. There is a need for reliable videos about adhesive capsulitis, with instructional and high-quality cited. In this way, patients can be directed to video sources with this quality video content.
