# Supplementary material for: The Lack of High-quality Educational Resources about Adhesive Capsulitis on YouTube
Source: Rev Bras Ortop (Sao Paulo). 2024 Apr 10;59(2):e260–8. doi: 10.1055/s-0044-1785465 (PMC11006515; doi:10.1055/s-0044-1785465)
Supplement: Related texts

# Research article: Ausência de recursos didáticos de alta qualidade sobre a capsulite adesiva no YouTube

**Keywords:** capsulite adesiva, bursite, gravação em vídeo, redes sociais, internet

## Abstract

**Objetivo** O advento da Internet proporcionou recursos novos e de fácil acesso para pacientes que procuram mais informações sobre saúde. Muitos médicos e organizações de saúde publicam vídeos informativos nesta plataforma e quase todos os pacientes procuram tais vídeos online para uma segunda opinião.

**Métodos** As frases “ *frozen shoulder* (ombro congelado)”, “ *frozen shoulder treatment* (tratamento de ombro congelado)”, “ *adhesive capsulitis* (capsulite adesiva)” e “ *adhesive capsulitis treatment* (tratamento de capsulite adesiva)” foram inseridas na barra de pesquisa do YouTube para uma consulta normal. A informatividade e a qualidade geral dos vídeos sobre capsulite adesiva foram avaliadas usando três escalas distintas.

**Resultados** Os valores de média e desvio padrão dos sistemas de pontuação do *Journal of the American Medical Association* (JAMA) foram 1,25 ± 0,51, DISCERN, 39,4 ± 13,4, *Global Quality Score* (GQS, Índice de Qualidade Global em português) 2,83 ± 0,96 e *Adhesive Capsulitis Specific Score* (ACSS, Escore Específico de Capsulite Adesiva em português), 7,43 ± 4,86, respectivamente. O número de visualizações, a taxa de visualizações e as curtidas tiveram uma correlação positiva com GQS, DISCERN e ACSS. Não houve diferença estatisticamente significativa entre os valores medianos de JAMA, GQS e DISCERN de acordo com a fonte/carregador do vídeo (p > 0,05).

**Conclusão** Os vídeos do YouTube sobre capsulite adesiva precisam ter maior qualidade, confiabilidade e qualidade instrutiva. Há necessidade de vídeos confiáveis sobre capsulite adesiva, com citações instrutivas e de alta qualidade.

## Introdução

O advento da Internet trouxe novos recursos novos facilmente acessíveis para os pacientes que procuram mais informações sobre saúde. 1 Nas pesquisas amplas na Internet, o YouTube fica atrás apenas do Google em popularidade. Os pacientes, no entanto, estão cada vez mais inclinados a buscar essas informações como forma de aprender sobre as opções terapêuticas à disposição. 2 Muitos médicos e organizações de saúde publicam vídeos informativos nesta plataforma e quase todos os pacientes procuram vídeos *online* para obter uma segunda opinião. O YouTube não é uma plataforma revista por pares e, assim, levanta questões sobre a confiabilidade das informações apresentadas em seus vídeos médicos. 3 A capsulite adesiva, também conhecida como ombro congelado, é um problema comum no ombro que se manifesta com perda progressiva de movimento glenoumeral e dor. 4 Essa doença é um dos problemas musculoesqueléticos mais comuns em ortopedia. Esta doença é bastante prevalente na população ortopédica. Apesar da prevalência deste problema e dos avanços na cirurgia do ombro, ainda há muitas questões o tratamento ideal. 5 Com estas incógnitas, é provável que os pacientes com capsulite adesiva usem o YouTube para explorar opções de tratamento.

Diversos estudos mostraram evidências de que a qualidade educacional dos vídeos do YouTube acerca de doenças ortopédicas é inadequada. 1 2 3 6 7 8 Apenas um estudo na literatura examina vídeos do YouTube relacionados à capsulite adesiva. 9 Os resultados deste estudo foram consistentes com os de outras pesquisas. No entanto, este estudo utilizou apenas vídeos relevantes para uma palavra-chave de pesquisa. 9 Nosso objetivo foi examinar o conteúdo informativo e a qualidade geral desses vídeos, expandindo as frases de pesquisa que pacientes com capsulite adesiva podem usar para encontrá-los no YouTube. Como em outros estudos na literatura, assumimos que as qualidades gerais e educacionais desses vídeos precisavam ser melhoradas.

## Materiais e Métodos

Em 18 de fevereiro de 2022, utilizando o Google Chrome (versão 92.0.4515.159-64 bits), com cache e cookies esvaziados, para uma busca no banco de dados do YouTube. Os assuntos foram “ *frozen shoulder* (ombro congelado)”, “ *frozen shoulder treatment* (tratamento de ombro congelado)”, “ *adhesive capsulitis* (capsulite adesiva)” e “ *adhesive capsulitis treatment* (tratamento de capsulite adesiva)” Foram incluídos os 50 principais vídeos para cada palavra-chave de pesquisa, escolhidos pelo algoritmo do YouTube com base na “relevância”. Isso rendeu um total de 200 vídeos para análise. 10 Os vídeos foram considerados para inclusão se atendessem aos seguintes critérios: eram m inglês, o assunto principal era o ombro congelado e a qualidade áudio e visual era satisfatória. Os vídeos foram excluídos se fossem repetitivos, não tivessem diálogo, estivessem em outro idioma que não o inglês, não tratassem de capsulite adesiva ou fossem categorizados como notícia, drama ou sátira. Não houve duração máxima para os vídeos e compilações de numerosos episódios foram contabilizadas como uma única obra. Uma conta no YouTube® foi criada para a pesquisa e, uma vez eliminadas as duplicatas, uma lista completa de URLs de vídeos foi compilada. Apenas 173 vídeos foram incluídos no estudo devido à exclusão de 26 que foram considerados repetitivos e um que estava em outro idioma que não o inglês.

Para cada vídeo do YouTube incluído, os seguintes atributos foram registrados: (1) título, (2) duração do vídeo, (3) visualizações, (4) fonte/carregador do vídeo, (5) tipo de conteúdo, (6) dias desde o upload, (7) taxa de visualização (visualizações/dia) e (8) curtidas. Os autores e carregadores dos vídeos foram classificados em sete grupos: (1) acadêmico (relacionado a autores ou carregadores afiliados a grupos de pesquisa, universidades ou faculdades), (2) médico (relacionado a médicos independentes ou grupos de médicos sem afiliação científica ou acadêmica), (3) não médico (profissionais de saúde que não fossem médicos licenciados), (4) instrutor, (5) fonte médica (conteúdo ou animações de *sites* de saúde), (6) paciente e (7) comercial. Os tipos de conteúdo foram categorizados como: (1) educação sobre exercícios, (2) informações específicas da doença, (3) experiência do paciente, (4) técnica ou abordagem cirúrgica, (5) manejo não cirúrgico e (6) publicidade.

Os critérios publicados no *Journal of the American Medical Association* (JAMA) foram utilizados para avaliar a precisão e confiabilidade dos vídeos ( Fig. 1 ). 11 Quatro fatores, cada um com peso 1, forneceram uma avaliação genérica da credibilidade do material citado. A precisão e a confiabilidade são melhor representadas por uma pontuação de 4, enquanto uma pontuação de 0 indica o oposto. Esses critérios têm sido amplamente utilizados na literatura para avaliar a confiabilidade de recursos *online* embora não terem sido validados. 10 12

**Fig. 1:** Critérios do *Journal of the American Medical Association* (JAMA).

Três escalas distintas avaliaram o valor educacional e a qualidade dos vídeos sobre capsulite adesiva. Cinco fatores calcularam o *Global Quality Score* (GQS, Índice de Qualidade Global em português) do conteúdo educacional ( Fig. 2 ). 10 13 A educação de qualidade é representada por uma pontuação máxima possível de 5. O *Adhesive Capsulitis Specific Score* (ACSS, Escore Específico de Capsulite Adesiva em português) foi desenvolvido para dados referentes à capsulite adesiva, fundamentados nas recomendações divulgadas pela *American Academy of Orthopaedic Surgeons* ( Fig. 3 ). O ACSS é um questionário de 21 itens que avalia informações sobre a apresentação e sintomas do paciente, capsulite adesiva em geral, procedimentos de diagnóstico e avaliação e opções terapêuticas à disposição. A maior qualidade é representada por uma pontuação mais alta, até no máximo 21. As faixas de possíveis classificações ACSS foram muito boa (21 pontos), boa (16 pontos), moderada (12 pontos), ruim (8 pontos) e muito ruim (4 pontos). 10 13 O escore DISCERN foi criado em Oxford, Reino Unido, para avaliação da qualidade de materiais escritos relacionados à saúde. É composto por 16 questões, cada uma com pontuação entre 1 e 5, dando um total possível de 6 a 80 pontos ( Fig. 4 ). 14 Nesta escala, as categorias de qualidade são péssima (16–28 pontos), ruim (29–41 pontos), regular (42–54 pontos), boa (55–67 pontos) e excelente (68–80 pontos).

**Fig. 2:** Escore global de qualidade.

**Fig. 3:** Escore específico de capsulite adesiva.

**Fig. 4:** Escore DISCERN.

Os vídeos incluídos no estudo foram determinados pelo autor não observador e apresentados aos observadores em formato de tabela. Os vídeos foram examinados e pontuados às cegas por dois observadores treinados em pontuação de pré-avaliação usando DISCERN, GQS, JAMA e ACSS. O coeficiente de correlação interclasses (ICC) determinou o nível de concordância entre os observadores; valores abaixo de 0,5 indicaram baixa confiabilidade, entre 0,5 e 0,75, confiabilidade moderada, entre 0,75 e 0,9, boa confiabilidade e, acima de 0,9, confiabilidade excelente.

O IBM SPSS Statistics versão 20 foi utilizado para a análise dos dados. Os dados contínuos foram resumidos como médias e desvios-padrão, enquanto os dados categóricos foram resumidos como porcentagens e frequências relativas. Os números foram arredondados em uma casa decimal. A confiabilidade e a qualidade do vídeo foram comparadas entre fontes e conteúdo de acordo com a análise de variância unidirecional (ANOVA) ou testes de Kruskal-Wallis, dependendo da distribuição dos dados. As diferenças entre os grupos foram examinadas com o teste U de Mann-Whitney para significância estatística. O nível de concordância entre os revisores foi determinado por meio de ICC. O coeficiente de correlação de Spearman examinou as correlações entre as avaliações da utilidade dos vídeos e suas características técnicas. A significância estatística foi determinada pelo valor de p inferior a 0,05.

## Resultados

As médias das características dos vídeos incluídos no estudo foram: duração do vídeo, 16,73 ± 123,09 minutos; número de visualizações, 264.431,7 ± 617.136,8, número de dias após o carregamento, 1.537,95 ± 1.159,3 dias; taxa de visualização, 269,75 ± 867,91; e número de curtidas, 3.826,78 ± 11.595,45. Quanto à distribuição da fonte/carregador de vídeo, 12 (6,9%) eram acadêmicos, 72 (41,6%) eram médicos, 71 (41%) eram não médicos, um (0,6%) era treinador, 13 (7,5%) eram fontes médicas, dois (1,2%) eram pacientes e dois (1,2%) eram comerciais. Observando o conteúdo dos vídeos, 44 (25,4%) se referiam a treinamento físico, 112 (64,7%), a informações específicas sobre a doença, três (1,7%) eram experiências de pacientes, 11 (6,4%) eram técnica/abordagem cirúrgica e três (1,7%) incluíam manejo não cirúrgico.

De acordo com os critérios JAMA, 95,9% dos vídeos receberam 2 pontos ou menos. No GQS, 27,7% dos vídeos foram avaliados com 2 pontos ou menos. Nos critérios DISCERN, 38 (21,9%) vídeos eram muito ruins, 47 (27,2%) eram ruins, 62 (35,9%) eram regulares, 22 (12,7%) eram bons e quatro (2,3%) eram muito bons. Segundo o ACSS, três (1,7%) vídeos eram muito bons, 31 (17,9%), bons, 37 (21,4%), regulares, 40 (23,1%), ruins e 62 (35,9%), muito ruins. Os valores de média e desvio padrão dos sistemas de pontuação foram JAMA, 1,25 ± 0,51; DISCERN, 39,4 ± 13,4; GQS 2,83 ± 0,96; e ACSS, 7,43 ± 4,86. Houve correlações positivas entre número de visualizações e GQS, taxa de visualização e GQS e entre curtidas e GQS, DISCERN e ACSS (r: 0,364, p < 0,001; r: 0,414, p < 0,001; r: 0,458, p< 0,001; r: 0,265, p < 0,001; r: 0,168, p < 0,027, respectivamente). Não houve diferença estatisticamente significativa entre os valores medianos de JAMA, pontuação GQS e critérios DISCERN de acordo com a fonte/carregador de vídeo (p > 0,05). Os valores dos sistemas de pontuação de acordo com a fonte/carregador de vídeo estão resumidos na Tabela 1 .

**Tabela 1:** Valores de média e desvio-padrão por fonte/carregador do vídeo

	Acadêmico	Médico	Não médico	Fonte médica
	Média ± DP	Média ± DP	Média ± DP	Média ± DP
JAMA	1,95 ± 1,15	1,19 ± 0,38	1,19 ± 0,41	1,26 ± 0,33
GQS	3,66 ± 0,65	2,45 ± 1	3,14 ± 0,75	2,96 ± 0,87
Critérios DISCERN	57 ± 14,37	36,74 ± 13,82	40,02 ± 10,37	40,88 ± 11,73
ACSS	13,45 ± 4,55	7,28 ± 5,13	9,53 ± 4,7	3,5 ± 3,53

DP, Desvio-padrão; JAMA, *Journal of the American Medical Association* ; GQS, *Global Quality Score* ; ACSS, *Adhesive Capsulitis Specific Score* .

Houve uma diferença estatisticamente significativa entre os valores medianos de ACSS de acordo com a fonte/carregador de vídeo (p = 0,013). Aqui, a diferença se referia aos valores medianos de ACSS de vídeos cuja fonte de carregamento era um instrutor e daqueles que eram de uma fonte médica. O valor mediano do ACSS da fonte de carregamento do vídeo foi 5, enquanto o valor mediano de PPIS foi 9 para a fonte médica ( Tabela 2 ). Não houve diferença estatisticamente significativa entre os valores medianos de JAMA, GQS, critérios DISCERN e ACSSS de acordo com o tipo de conteúdo ( Tabela 3 ).

**Tabela 2:** Comparação dos escores por fonte/carregador do vídeo

Fonte/carregador do vídeo								
	Acadêmico	Médico	Não Médico	Treinador	Fonte médica	Paciente	Comercial	Valor d p *
	Média (min-max)	Média (min-max)	Média (min-max)	Média (min-max)	Média (min-max)	Média (min-max)	Média (min-max)	
JAMA	1,5 (1-4)	2 (1-3)	1,5 (1-4)	1 (1-3)	2 (1-3)	1 (1-1)	1 (1-4)	0,081
GQS	2 (1-4)	2,8 (1-5)	2,5 (1-5)	2,5 (1-4)	3 (1-5)	1,8 (1-2,5)	2,5 (1-4)	0,715
Critérios DISCERN	24 (16-47,5)	36 (14,5-56)	34 (15,5-55)	38 (16-63,5)	42 (23,5-54)	22 (14-30)	34 (20-46)	0,065
ACSS	5 (2-10) ^ab^	6,3 (1,5-17,5) ^ab^	6 (1-18) ^ab^	5 (1-13,5) ^a^	9 (5-14,5) ^b^	3 (2-4) ^ab^	9 (4-16) ^ab^	0,013

* Teste de Kruskal Wallis; ^a-b^ : Ausência de diferença entre grupos com a mesma letra.

Min, mínimo; max, máximo; JAMA, *Journal of the American Medical Association* **;** GQS, *Global Quality Score* ; ACSS, *Adhesive Capsulitis Specific Score* .

**Tabela 3:** Comparação das fontes por tipo de conteúdo

	Tipo de conteúdo					
Educação sobre exercício	Informações específicas sobre a doença	Experiência do paciente	Técnica ou abordagem cirúrgica	Manejo não operatório	Propaganda	Valor de p *
Média (min-max)	Média (min-max)	Média (min-max)	Média (min-max)	Média (min-max)	Média (min-max)	
JAMA 1,5 (1-3)	2 (1-4)	1 (1-1)	1,5 (1-4)	1,5 (1-3,5)	1 (1-4)	0,191
GQS 3 (1-4)	2,5 (1-4,5)	2,5 (2,5-2,5)	3 (1-5)	2,5 (1-5)	3 (1-4)	0,934
Critérios DISCERN 37,8 (25-63,5)	36,5 (15-55)	30 (30-30)	33 (14,5-56)	35 (14-54,5)	35 (20-46)	0,483
ACSS 5 (1-9)	7 (1,5-18)	4 (4-4)	6 (2,5-12,5)	5,5 (1-14,5)	9,5 (4-16)	0,051

* Kruskal Wallis Testi.

Min, mínimo; max, máximo; JAMA, *Journal of the American Medical Association* **;** GQS, *Global Quality Score* ; ACSS, *Adhesive Capsulitis Specific Score* .

## Discussão

As conclusões essenciais deste estudo estão de acordo com os critérios JAMA, já que 95,9% dos vídeos foram avaliados com 2 pontos ou menos. De acordo com GQS, 27,7% dos vídeos receberam 2 pontos ou menos. Segundo os critérios do DISCERN, 49,1% dos vídeos foram muito ruins ou ruins. O ACSS classificou 59% dos vídeos como ruins ou muito ruins. Esses achados são semelhantes aos de Tang et al., 9 que avaliaram o ensino e a qualidade dos vídeos sobre capsulite adesiva. Este estudo analisou a confiabilidade do vídeo com pontuação JAMA e mais termos de pesquisa e vídeos de capsulite adesiva que os pacientes podem pesquisar no YouTube. Outra característica deste estudo é que não há restrição de duração do vídeo porque a maior duração dos vídeos aumenta seu conteúdo informativo e educativo. 8 A não avaliação de vídeos longos pode afetar os resultados da pesquisa ao excluir vídeos altamente educativos. Como resultado da avaliação abrangente, este estudo concluiu que a confiabilidade, a qualidade e o nível educacional dos vídeos do YouTube relacionados à capsulite adesiva precisam ser melhorados.

Os vídeos enviados ao YouTube não passam por processo de avaliação. 3 Por isso, o número de curtidas e visualizações dos vídeos pode criar uma percepção de qualidade pelos pacientes e gerar desinformação. 15 Consequentemente, o número de visualizações dos vídeos considerados benéficos para os pacientes pode ser menor. 16 17 Neste estudo, quanto ao número de curtidas e a pontuação, houve correlação positiva entre o número de visualizações e GQS. Esses achados mostram que os pacientes tendem a assistir vídeos sobre capsulite adesiva de melhor qualidade e gostam daqueles que são altamente educativos. Nossos achados podem ser interpretados como pacientes com capsulite adesiva preferem vídeos educativos e de alta qualidade, mas o que número desses vídeos é insuficiente.

As instruções para vídeos do YouTube podem variar dependendo do remetente do vídeo e da fonte. 18 Koller et al. 18 avaliaram vídeos sobre artrite de quadril e descobriram que aqueles de origem acadêmica e médica são mais educativos. No entanto, neste estudo, fontes médicas ou acadêmicas não forneceram mais informações educacionais do que outros carregadores. Vídeos com fins e preocupações comerciais podem ter consequências negativas no tratamento dos pacientes. 19 A principal causa de vídeos de má qualidade pode estar relacionada às questões comerciais. Dado que a maioria dos filmes são feitos conforme a prática do fornecedor e não há obrigação de responsabilidade médico-paciente, a maioria dos fornecedores pode sentir-se à vontade para aconselhar o público apenas sobre partes específicas da doença e dos métodos de tratamento. 3 Isto pode fazer com que os pacientes com capsulite adesiva tendam a afirmar que o único método de tratamento oferecido é a opção certa e solicitar o tratamento errado. A solução para esta situação pode passar por preparar plataformas de informação dos pacientes sem preocupações comerciais e encaminhar os pacientes para essas plataformas.

Pacientes jovens usam muitas plataformas de mídia social além do YouTube para aprender sobre sua doença. 20 Agentes de conversação de inteligência artificial (IA) (ou *chatbots* ) têm se mostrado promissores como envolvimento direto do paciente e ferramentas para educação; o Chat GPT é um exemplo. 21 Hoje em dia, algoritmos de IA com linguagem natural são feitos para absorver dados que não estão bem-organizados ou padronizados e, então, fornecer resultados que parecem humanos. Esses algoritmos baseiam-se em um grande corpo de material previamente escrito por humanos para criar respostas que tenham alta probabilidade de corresponder à consulta do usuário. Os *chatbots* podem melhorar os cuidados médicos, fornecendo respostas instantâneas às preocupações dos pacientes, mas como são treinados em padrões de linguagem em vez de bases de dados objetivas, correndo o risco de dar aos pacientes respostas erradas, mas aparentemente confiáveis. 22 Para aprender mais sobre o capacidade e valor educacional das informações que os pacientes acessam sobre ombro congelado na internet, mais dados podem ser obtidos por meio de estudos que examinam o ombro congelado em diferentes plataformas de mídia social. Além disso, há necessidade de avaliar as informações que os *chatbots* fornecem aos pacientes sobre o ombro congelado. Considerando esses dados, modelos de IA podem ser treinados por médicos. Dessa forma, apresentações de *slides* e vídeos com informações precisas e confiáveis para os pacientes podem ser preparados com suporte da IA e disponibilizadas aos pacientes.

Este estudo tem algumas limitações. Os vídeos são continuamente adicionados ao YouTube, tornando-o uma plataforma dinâmica. O YouTube também oferece vídeos personalizados com IA. Portanto, os vídeos assistidos nas buscas podem refletir apenas parcialmente os vídeos apresentados aos pacientes. Usamos *software* de provedor de Internet com cookies e histórico limpos para minimizar a apresentação personalizada de vídeos. No entanto, estudos anteriores também utilizaram esse método. 12 23 Novamente, usar apenas vídeos em inglês e pesquisar somente em um local pode alterar as propriedades dos vídeos avaliados. A IA pode oferecer vídeos diferentes de acordo com países e localidades. Termos de pesquisa e vídeos em outros idiomas além do inglês podem ter conteúdos informativos diferentes. Apenas os primeiros 50 vídeos de cada termo de pesquisa foram avaliados. Os vídeos avaliados representam uma pequena fração dos vídeos sobre capsulite adesiva. Os resultados podem mudar com o aumento dos vídeos avaliados. No entanto, esse método já foi utilizado. 10 12 Ao mesmo tempo, embora tenhamos avaliado os vídeos de capsulite adesiva expandindo os termos de pesquisa neste estudo, assumimos que obtivemos dados semelhantes aos de Tang et al. 9

A internet tornou mais fácil do que nunca o acesso a informações sobre qualquer assunto imaginável. No entanto, isso também significa que muitas informações e desinformações estão disponíveis *online* . Isto pode ser um problema, pois as pessoas podem não conseguir distinguir entre informações confiáveis ou não. Uma forma de resolver esse problema é filtrar as informações na internet. Isso pode ser feito usando programas que identificam e bloqueiam o conteúdo prejudicial ou enganoso. No entanto, é importante notar que nenhum sistema de filtragem é perfeito e algumas informações falsas ou enganosas ainda podem escapar. Outra forma de resolver o problema é educar as pessoas na avaliação crítica da informação. Isto inclui ensinar as pessoas a identificarem as fontes de informação confiáveis, identificar vieses e avaliar a qualidade das evidências. Também é importante estar ciente das limitações da internet. A internet é um recurso vasto e em constante mudança e pode ser difícil acompanhar todas as novas informações publicadas. Isso significa que é importante ser cético em relação às informações encontradas *online* e sempre fazer sua própria pesquisa antes de tirar qualquer conclusão. Aqui estão algumas dicas para avaliar informações na internet: Considere a fonte da informação. É um *site* ou organização confiável? Procure evidências para apoiar as afirmações feitas. Há algum estudo ou estatística citada? Esteja ciente dos vieses. A informação vem de uma fonte tendenciosa, como um partido político ou um grupo de interesse especial? Use seu bom senso. Se algo parece bom demais para ser verdade, provavelmente é. Seguindo essas dicas, você pode ajudar a garantir a obtenção de informações precisas e confiáveis da internet.

Considerando esses achados, há necessidade de vídeos educativos e de alta qualidade para informação dos pacientes. Deve haver vídeos claros e de alta qualidade que tratem do ombro congelado como um todo, preparados por cirurgiões de ombro e suas associações. Esses vídeos devem ser enviados para *sites* públicos e os pacientes devem ser direcionados para esses vídeos. O preparo desses vídeos pode ser beneficiado por informações em https://www.mayoclinic.org/diseases-conditions/frozen-shoulder/symptoms-causes/syc-20372684 , https://www.healthline.com/health/frozen-shoulder e https://orthoinfo.aaos.org/en/diseases-conditions/frozen-shoulder/ . Além disso, ao treinar *software* de inteligência artificial nesta doença, muitos vídeos com conteúdo de alta qualidade sobre ombro congelado podem ser preparados de forma rápida e eficaz e disponibilizados aos pacientes.

## Conclusão

Os vídeos do YouTube sobre capsulite adesiva, portanto, precisam ser de maior qualidade, confiabilidade e qualidade instrutiva. Há necessidade de vídeos confiáveis sobre capsulite adesiva, com citações instrutivas e de alta qualidade. Desta forma, os pacientes podem ser direcionados para fontes de vídeo com conteúdo de vídeo de qualidade.
